# A Potassium Ion-Exchanged Glass Optical Waveguide Sensor Locally Coated with a Crystal Violet-SiO_2_ Gel Film for Real-Time Detection of Organophosphorus Pesticides Simulant

**DOI:** 10.3390/s19194219

**Published:** 2019-09-28

**Authors:** Bin Du, Zhaoyang Tong, Xihui Mu, Jianjie Xu, Shuai Liu, Zhiwei Liu, Wei Cao, Zhi-Mei Qi

**Affiliations:** 1 State Key Laboratory of NBC Protection for Civilian, Beijing 102205, China; dubin51979@163.com (B.D.); muxh0511@163.com (X.M.); juechen1981@163.com (J.X.); 15155922415@163.com (S.L.); liuzhiwei20190901@163.com (Z.L.); odysseus1988@126.com (W.C.); 2 State Key Laboratory of Transducer Technology, Institute of Electronics, Chinese Academy of Sciences, Beijing 100190, China; zhimei-qi@mail.ie.ac.cn

**Keywords:** optical waveguide, organophosphorus pesticides, crystal violet, sensor, detection

## Abstract

An optical waveguide (OWG) sensor was developed for real-time detection of diethyl chlorophosphate (DCP) vapor, which is a typical simulant for organophosphorus pesticides and chemical weapon agents. Silica gel, crystal violet (CV), and potassium ion-exchange (PIE) OWG were used to fabricate the sensor’s device. In the real-time detection of the DCP vapor, the volume fraction of DCP vapor was recorded to be as low as 1.68 × 10^−9^. Moreover, the detection mechanism of CV-SiO_2_ gel film coated the PIE OWG sensor for DCP, which was evaluated by absorption spectra. These results demonstrated that the change of output light intensity of the OWG sensor significantly increased with the augment of the DCP concentration. Repeatability as well as selectivity of the sensors were tested using 0.042 × 10^−6^ and 26.32 × 10^−6^ volume fraction of the DCP vapor. No clear interference with the DCP detection was observed in the presence of other common solvents (e.g., acetone, methanol, dichloromethane, dimethylsulfoxide, and tetrahydrofuran), benzene series (e.g., benzene, toluene, chlorobenzene, and aniline), phosphorus-containing reagents (e.g., dimethyl methylphosphonate and trimethyl phosphate), acid, and basic gas (e.g., acetic acid and 25% ammonium hydroxide), which demonstrates that the OWG sensor could provide real-time, fast, and accurate measurement results for the detection of DCP.

## 1. Introduction

Large-scale abuse of organophosphorus pesticides for decades has caused serious pollution in the environment. The toxicity and refractory degradation of traditional pesticides have caused serious threats to the human body and ecology. Therefore, the detection of pesticides has attracted the interest of many researchers. Due to the high toxicity of pesticides, some comparatively innocuous and readily available organophosphorus compounds, have been used as alternative safe simulants in experimental research studies for the detection of phosphorus containing agents. Diethyl chlorophosphate (DCP) is a pharmaceutical intermediate with low toxicity and is often used as a simulant for pesticides. The phosphoryl chloride group of DCP is similar to Sarin’s phosphoryl fluoride group because it has an important practical value as a safe alternative mimic for chemical weapon agents [1,2,3]. Instrumental analyses such as chromatography [4,5], ion migration spectrometry [6,7,8], Raman [9,10,11], etc., are frequently applied, but these approaches show at least one of the following limitations: high-cost, pre-treatment, and operational complexity. Fluorescent [12,13,14] and colorimetric [15,16] methods have also been developed. Normally, they have not yet met the demands of sensitive, real-time, and quantitative detection of gaseous analytes. Therefore, there is an urgent need to develop a new method for on-site and low-cost detection of organophosphorus pesticides and their simulants.

**Scheme 1:**
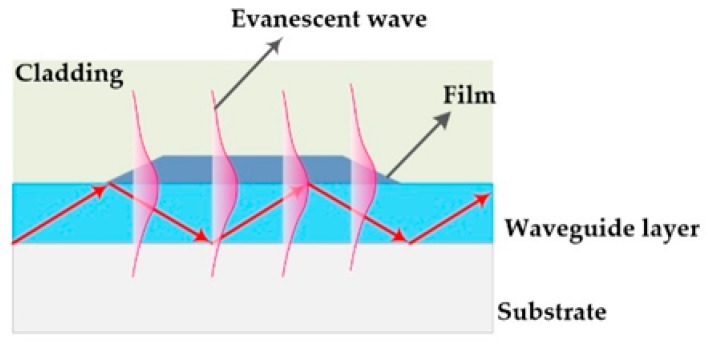
Schematic diagram of optical waveguide and evanescent wave.

Optical waveguide (OWG) is an optical structure that has been used in fabricating optical devices in numerous fields such as the optical sensor [17,18]. OWG sensors obtained by fabricating a sensitive film on the waveguide layer of the OWG is very sensitive to changes in cladding’s physical and optical properties, such as refractive index, absorbance, density, and the like. Therefore, the evanescent wave generated at the interface between the waveguide layer and the sensitive film by the laser, which transmitted in the waveguide layer at total reflection mode can be used as a probe to detect the analyte (Scheme 1). OWG sensors own the advantages of high sensitivity, fast response time, and system compactness, and they have witnessed widespread application in the detecting cell [19,20,21], biological molecules [22,23], and metal ions [24], as well as in the detection of low-concentration gas (such as xylene [25,26], chlorobenzene [27], ammonia [28], trimethylamine [29], and hydrogen chloride [30]). Inspired by these advantages, we developed an OWG sensor that is low-cost and has both a real-time and sensitive detection DCP vapor.

Silica gel film has received extensive attention due to their non-toxicity, low density, high porosity, and high specific surface area, and can be conveniently prepared by the sol-gel method [31]. Such a network-like silica gel film has significant application prospects in the fields of structural materials and optical materials. For its excellent optical properties, silica gel film could be used as sensitive film of OWG and film-forming material of sensitive molecular. As a kind of triphenylmethane dyes, crystal violet (CV) can be used as not only an acid-base indicator, but also a skin disinfectant. CV is a biological stain and ion association agent that can be used for photometric determination of metal ions. To date, there are few reports on the use of CV for pesticide detecting.

In this study, silica gel film was formed by hydrolysis of tetraethoxysilane (TEOS), and CV-SiO_2_ gel film coated potassium ion-exchange (PIE). OWG was fabricated by spin-coating a silica gel doped with CV on a glass optical waveguide. Then the OWG sensor was established by placing CV-SiO_2_ gel film coated PIE OWG chip on the gas testing system. At last, the DCP vapor was tested by the OWG sensor. The sensing mechanism was investigated and its reproducibility and selectivity were also discussed. Meanwhile, the sensitive film without silica gel was also fabricated, and the performance of CV coated PIE OWG chip was also studied.

## 2. Materials and Methods

### 2.1. Materials and Instruments

Crystal violet, pure, indicator, was obtained from Acros Organics (Geel, Belgium). Diethyl chlorophosphate (97%) and Tetraethoxysilane (98%), which was used for preparing silica sol, were purchased from Alfa Aesar (Tewksbury, MA, USA). Diiodomethane (CH_2_I_2_, n = 1.74) was received from J&K Scientific Ltd. (Beijing, China). All the other reagents were of analysis grade and purchased from Beijing Chemical Works (Beijing, China).

Micro slide glass (S1111, 76 mm × 26 mm in area, and 1.0 mm in depth, Matsunami Glass Ind., Ltd., Osaka, Japan) with a refractive index of 1.52 was adopted as the single mode planar waveguide. PIE OWGs were fabricated in a ceramic fiber muffle furnace (MF-0610F, Huagangtong Technology (Beijing) Co., Ltd., Beijing, China). The thin film was coated onto the surface of PIE OWG using a spin coater (KW-4A, Institute of Microelectronics of the Chinese Academy of Sciences, Beijing, China). The topographies were characterized by a scanning probe microscope (SPM) (SOLVER NEXT, NT-MDT Co., Moscow, Russia). Absorption spectra were determined on BioMATE 3S UV-Vis spectrophotometer (Thermo Fisher Scientific Inc., Waltham, MA, USA). A gas sampler introduced the samples (QC-1S, Beijing Municipal Institute of Labor Protection, Beijing, China).

### 2.2. K^+^ Ion-Exchanged Glass OWG Preparation

Single mode planar waveguides were prepared on soda-lime glass substrates by the K^+^-Na^+^ ion-exchange method [32]. KNO_3_ was annealed at 400 °C and melted in a ceramic fiber muffle furnace. Then, a dry micro slide was immersed in the molten KNO_3_ (maintained at 400 °C) for 40 min. The as-obtained glass OWG was cooled to room temperature, and washed with distilled water. Then it was dried afterward.

### 2.3. Fabrication of the CV-SiO_2_ Gel Film Coated PIE OWG Chip

To obtain CV-SiO_2_ sol mixed solution, 20 mg of CV was added to 9 mL TEOS and 9 mL ethanol mixture. After stirring, 3 mL distilled water was slowly added dropwise and stirred at room temperature for 1 h to obtain a uniform and transparent violet solution. It was coated on the surface of the PIE OWG by using the spin-coating method to obtain a CV-SiO_2_ gel film coated PIE OWG chip. The first rotating speed of the spin coater was 500 rpm, with a rotating time of 10 s, and the second rotating speed was 3000 rpm, with a rotating time of 30 s. Then the coated waveguide was baked for 2 h at 60 °C.

As a contrast, the sensitive film without silica gel was also fabricated. In short, 20 mg of CV was dissolved in an 18 mL ethanol mixture. After stirring, 3 mL distilled water was slowly added dropwise and stirred at room temperature for 1 h to obtain a uniform and transparent violet solution. In addition, the CV coated PIE OWG chip was fabricated following the above process.

### 2.4. Measurement Procedures for Testing for Gas

In this article, different concentrations of standard DCP vapor were prepared by the following steps. In the first place, standard DCP vapor was obtained by vaporizing a quantity of 97% DCP inside a 500 mL standard vessel. Then different amounts of standard DCP vapor from the first vessel were injected into the second standard 500 mL vessel to obtain the desired concentrations by syringe and then diluted with dry air. Much lower concentrations of DCP (in the ppm level) could be obtained by using this standard vessel dilution method. A new glass syringe was used to inject the same volume of the DCP sample into the sample chamber. The tee valve was turned to the gas circuit of the analyte vapor when the measurements were carried on, and then turned to the gas circuit of air after the analyte test to evacuate the DCP vapor. Meanwhile, by extracting the gas above the surface of the liquid, saturated steam of other common solvents, benzene series, and phosphorus-containing reagents were obtained and used for the study of the sensor’s selectivity. All the measurements were carried out at room temperature.

**Scheme 2:**
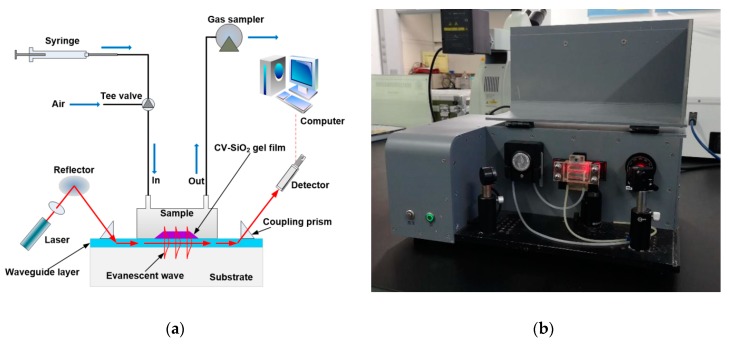
Schematic diagram of the OWG sensor (**a**) and photography of the sensor (**b**).

The gas testing system, shown in Scheme 2, was constructed by a sample syringe containing DCP vapor with a certain concentration, gas sampler, CV-SiO_2_ gel film coated PIE OWG chip, sample chamber, 633 nm laser source, tee valve, reflector, photodetector, and computer. The CV-SiO_2_ gel film coated PIE OWG chip was placed on the back board of the sample chamber, and the sample chamber attached to the middle of the sensing chip to cover the entire CV-SiO_2_ gel film for sensing performance. Two glass prisms (*n* = 1.799) were mounted at each end of the sensing chip. The 633 nm beam was introduced into the waveguide layer of the OWG sensing chip via a prism coupling method to excite the evanescent wave, and it emerged from another prism coupler. To increase the coupling efficiency, diiodomethane (*n* = 1.74) was introduced between the prisms and the waveguide as the index-matching liquid. The output light signals were monitored and converted into electric signals by a photodetector and recorded by the computer.

## 3. Results and Discussion

### 3.1. Topographical Characterization of the CV-SiO_2_ Gel Film

**Figure 1:**
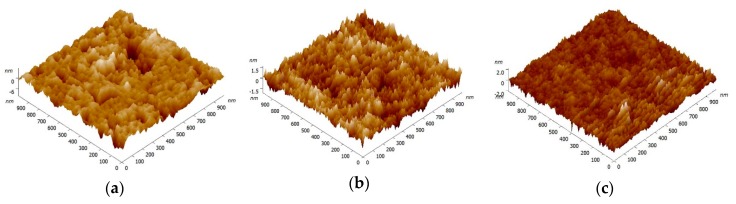
AFM images (1 μm × 1 μm) of glass substrate (**a**), CV-SiO_2_ gel film (**b**), and SiO_2_ gel film (**c**).

**Figure 2:**
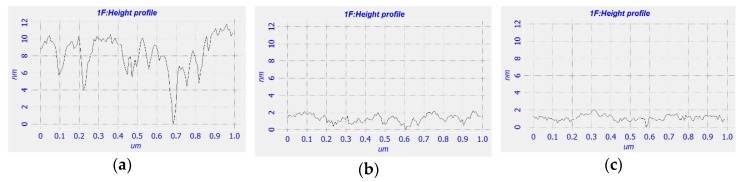
Section analysis of glass substrate (**a**), CV-SiO_2_ gel film (**b**), and SiO_2_ gel film (**c**).

**Figure 3:**
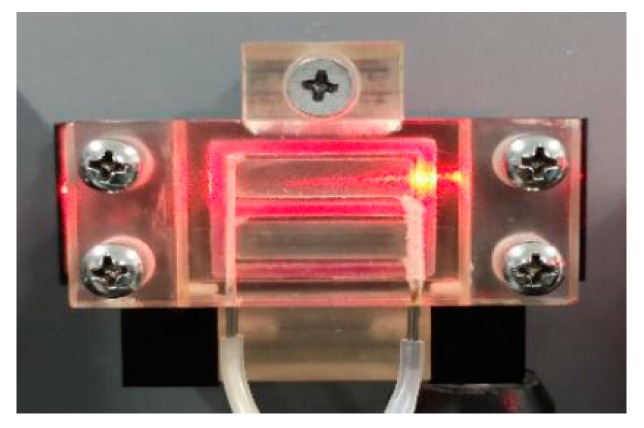
Photograph of a scattering-induced streak along the traveling path of the 633 nm laser in the CV-SiO_2_ gel film.

The surface morphology and topography of CV-SiO_2_ gel film was obtained by a SOLVER NEXT SPM. As a comparison, atomic force microscope (AFM) images of the glass substrate and SiO_2_ gel film were also given in Figure 1. Figure 1a clearly shows the rough appearance of the original glass substrate with rule-less particle distribution, and its root-mean-square (RMS) roughness was approximately 1.664 nm. In contrast, the appearance of the CV-SiO_2_ gel film was dense and relatively smooth with nearly no particle. We could also see a decline in RMS roughness to 0.5 nm (Figure 1b). Meanwhile, Figure 1c demonstrated that the morphology of SiO_2_ gel film was similar to the CV-SiO_2_ gel film with RMS roughness of 0.43 nm, which indicated that CV was uniformly dispersed in the silica gel film. The section analysis of the glass substrate, CV-SiO_2_ gel film, and SiO_2_ gel film were also exhibited in Figure 2, which clearly show that surfaces of CV-SiO_2_ gel film and SiO_2_ gel film were smoother than the glass substrate. All these topographical characterizations show that an excellent optical film with smooth morphology was obtained by spin coating (Figure 3).

### 3.2. Performance of the Sensor Device

**Figure 4:**
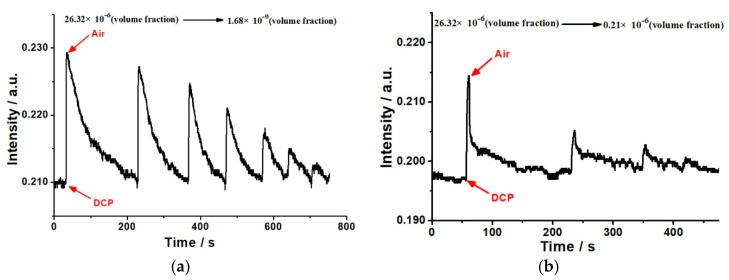
Typical response of CV-SiO_2_ gel film coated PIE OWG sensor (**a**) and CV coated PIE OWG sensor (**b**) when exposed to DCP vapor.

The reversible response of the CV-SiO_2_ gel film-coated PIE OWG sensor to various concentrations of the DCP vapor is shown in Figure 4a. It can be observed that, when the CV-SiO_2_ gel film is exposed to DCP vapor, the output light intensity (signal) increased rapidly. Conversely, the signal completely returned to its initial level when dry air was introduced afterward to replace the DCP vapor. The output light intensity (*I*) increased with the rise of the DCP vapor’s concentration in the range of 1.68 × 10^−9^~26.32 × 10^−6^ (volume fraction) and decreased when its concentration was reduced. In contrast with CV-SiO_2_ gel film, the performance of CV-coated PIE OWG sensor was also evaluated (Figure 4b). Similar to CV-SiO_2_ gel film, the injection of DCP vapor could also change the *I* of CV coated PIE OWG sensor, but its quantitative limit was much higher than the CV-SiO_2_ gel film coated PIE OWG sensor. In addition, it is worth noting that it took a shorter time for this sensor to return to its primary level by the means of dry air. The possible reason is that DCP vapor could be exhausted from the CV film faster than that of CV-SiO_2_ gel film, and this phenomenon also demonstrates that silica gel film prepared by the sol-gel method possesses the multi-aperture structure. This increases the gas diffusion efficiency, and ensures that the OWG sensor possesses a lower quantitative limit.

**Figure 5:**
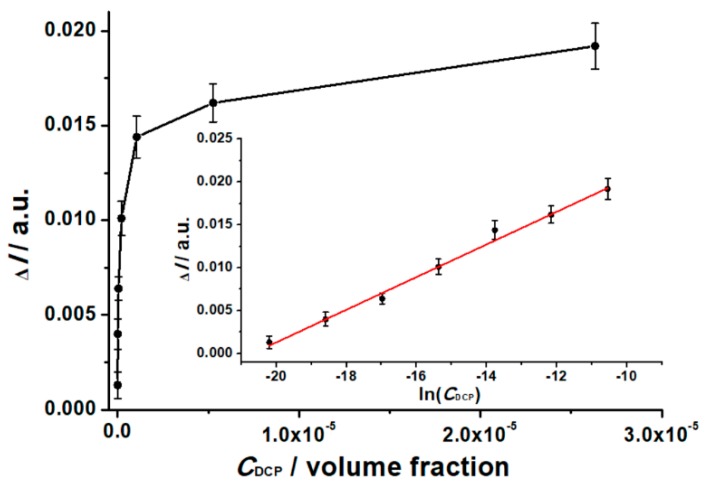
Relationship between the changes of output light intensity of CV-SiO_2_ gel film coated the PIE OWG sensor and the concentration of the DCP vapor. Insert graph is the calibration curve.

The dynamic curve of the concentration of the DCP vapor (*C*_DCP_) and the change of OWG sensor’s output light intensity (_Δ_*I*) is shown in Figure 5. On the ordinate, the change of OWG sensor’s output light intensity is defined as _Δ_*I* = *I*_DCP_ − *I*_0_, where *I*_0_ is the initial output light intensity and *I*_DCP_ is the output light intensity at the highest point when the DCP vapor was injected into the sample chamber, respectively. From the insert graph of Figure 4, a strong dependence of CV-SiO_2_ gel film coated PIE OWG sensor’s output signal. _Δ_*I* was found to be linear to ln(*C*_DCP_)in the range of 1.68 × 10^−9^~26.32 × 10^−6^ (volume fraction), and the following relation was obtained: _Δ_*I* = 0.0019 ln(*C*_DCP_) + 0.03939 (*R*^2^ = 0.98998).

### 3.3. Sensing Principle

Regarding the OWG sensor, the sensing film’s optical properties (absorbance, scattering, refractive index, and fluorescence/luminescence) are the main factors that affect its sensitivity. When a thin film is coated on the surface of the waveguide layer, the evanescent wave, which was produced by the 633 nm light wave traveling through the waveguide layer, comes into the thin film. The change of OWG sensor’s output light intensity, which was caused by the variation of optical properties, is related to the analyte concentration, and this provides the foundation for the sensor response. In this paper, the variation in CV-SiO_2_ gel film’s absorbance/transmittance induced the changes in intensity of the output light when the sensing device was exposed to the DCP vapor.

To investigate the sensing principle, the changes in color and absorption spectra of CV solution and CV-SiO_2_ gel film before and after reacting with DCP were determined.

**Figure 6:**
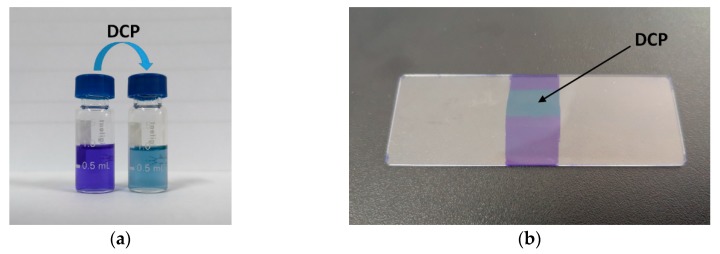
Color change of the CV solution (**a**, 2 μg/mL) and CV-SiO_2_ gel film (**b**).

As shown in Figure 6a, when DCP was added into CV in the ethanol solution, the color of the CV solution changed from violet to blue-gray. Similarly, when the CV-SiO_2_ gel film was exposed to DCP vapor, its color became blue-gray (Figure 6b).

**Figure 7:**
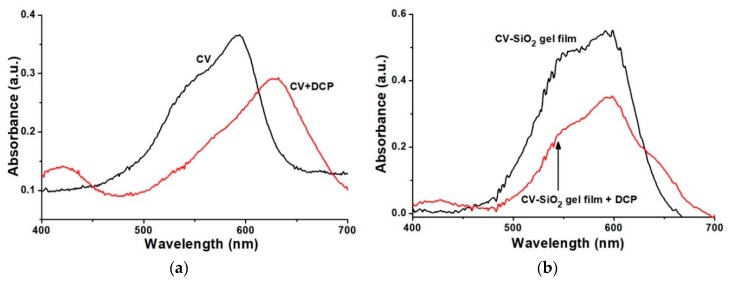
Absorbance change of the CV solution (**a**) and CV-SiO_2_ gel film (**b**).

The absorption spectra of 1 mL solution of CV in ethanol in a quartz cell before and after adding DCP were determined. The slides coated with CV-SiO_2_ gel film were placed and fixed inside the quartz cell and injected with a certain amount of DCP vapor, sealed the lid of the quartz cell to make sure DCP reacts with the film sufficiently, and then its absorbance at 633 nm was monitored. All the results are presented in Figure 7. It can be seen from Figure 7a that, when DCP was added into CV in the ethanol solution, bathochromic shift was observed in the absorption spectra and its absorbance at 633 nm significantly increased. Likewise, the similar phenomenon was noted after the CV-SiO_2_ gel film reacted with DCP vapor (Figure 7b). These changes in color and absorption spectra might be induced by the phosphorylation-reaction between the electron-deficient phosphorus in DCP and the nucleophilic moiety in CV, which resulted in ionization. This led to a change in the absorption spectrum, and influenced the sensor’s *I*. This principle was consistent with previous reports [33,34,35,36].

For a thin film, the relationship between absorbance *A* and absorption coefficient *α* is given by the Lambert-Beer law.

(1) A=αLC,

where *L* is the thickness of thin film, and *C* represents the concentration of CV in thin film. The absorption spectra of the film indicate that the adsorption and reaction between CV-SiO_2_ gel film and DCP vapor lead to an enhancement in the thin film’s absorbance *A* at 633 nm and an increase in the absorption coefficient *α*. The increase in thin film’s absorption coefficient *α* led to a decrease in the output light intensity of OWG.

Moreover, according to the Kramers-Kronig relationship [37], the following equation is true.

(2) $$n\left(\omega \right)=1+\frac{c}{\pi }℘\overset{+\infty }{\underset{0}{\int }}\frac{\alpha \left(\Omega \right)}{\Omega^{2}-\omega^{2}}d\Omega ,$$

where *n* is the refractive index of the CV-SiO_2_ gel film, *ω* is angular frequency, and ℘ represents the Cauchy Principal Value. When the CV-SiO_2_ gel film was exposed to the DCP vapor, its light absorbance characteristics and *n* changed, which affected the evanescent wave that penetrated into the sensitive film, and then influenced the output light intensity.

According to Kramers-Kronig relationship, the increase in thin film’s absorption coefficient *α* caused a decrease in the refractive index *n*, and it also weakened the evanescent field intensity inside the thin film, which reduced the absorption of the guided wave by the thin film. Therefore, an increase in the OWG’s output light intensity was observed.

The increase in the output light intensity of the OWG measured by the experiment indicated that there is a competitive relation between the absorption coefficient *α* and the refractive index *n* in the OWG’s output light intensity, and the refractive index *n* dominates.

### 3.4. Reproducibility and Selectivity of the Response of the OWG Sensor

**Figure 8:**
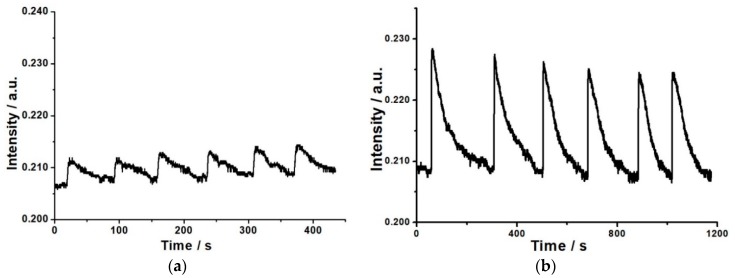
The repeatable response to 0.042 × 10^−6^ (**a**) and 26.32 × 10^−6^. (**b**) Volume fraction of DCP vapor.

Meanwhile, we tested the reversibility and stability of the CV-SiO_2_ gel film coated PIE OWG sensor. Figure 8 showed OWG sensor’s response to DCP vapor with a low concentration (0.042 × 10^−6^ (volume fraction)) and a high concentration (26.32 × 10^−6^ (volume fraction)). As can be seen from Figure 8a, dry air and 0.042 × 10^−6^ volume fraction of DCP vapor were sequentially injected into the sample chamber, and the cycle was repeated six times. When the DCP vapor was injected into the sample chamber, the output light intensity increased rapidly. Then the dry air was introduced afterward to supplant the DCP vapor. The output light intensity dropped to a primary level. Each measuring cycle was reversible during several consecutive cycles and the _Δ_*I* were substantially similar in the six cycles. Good reversibility indicates the binding of DCP vapor was reversible. The response time was rapid (5 s), and fully reversible (60 s) after six consecutive injections of 0.042 × 10^−6^ volume fraction of DCP vapor. At this concentration, the relative standard deviation (R.S.D) of the _Δ_*I* was 13.99%. Reversibility of the OWG sensor to 26.32 × 10^−6^ volume fraction DCP vapor was further studied (Figure 8b). At this concentration, its response and recovery time was 5 s and 180 s, respectively, and the R.S.D of the _Δ_*I* was 6.58%. These results demonstrated that the OWG sensor response was fully reproducible and reversible for DCP vapor detection.

**Figure 9:**
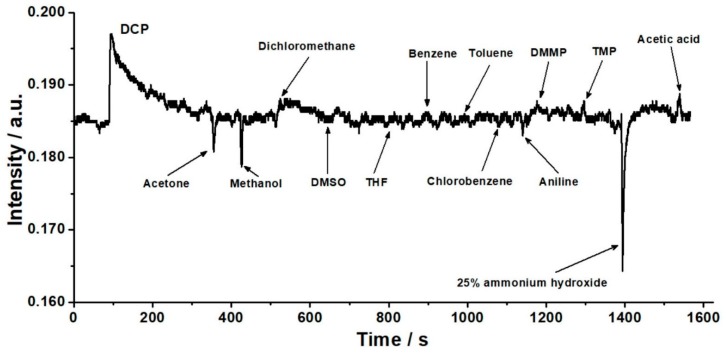
Selective response to saturated steam of various gases and 26.32 × 10^−6^ volume fraction of the DCP vapor.

On account of the presence of interferents in practical operation conditions, such as common solvents and benzene series in pesticide spraying, may interfere with the results, the selectivity of CV-SiO_2_ gel film-coated PIE OWG sensor to various gases was evaluated. The response of the OWG sensor to 20 mL of saturated steam of other common solvents (acetone, methanol, dichloromethane, dimethylsulfoxide (DMSO), and tetrahydrofuran (THF)), benzene series (benzene, toluene, chlorobenzene, and aniline), acid, and basic gas (acetic acid and 25% ammonium hydroxide) were recorded. Meanwhile, typical phosphorus-containing reagents such as dimethyl methylphosphonate (DMMP) and trimethyl phosphate (TMP) were selected to test its selectivity. As shown in Figure 9, the output light intensity dropped when acetone, methanol, and 25% ammonium hydroxide were injected into the sample chamber, and only acetic acid could induce a slight increment of the output light intensity. The OWG sensor did not exhibit a meaningful response to all the other common solvents, benzene series, and phosphorus-containing reagents mentioned above. On the basis of these facts, we can assume that the CV-SiO_2_ gel film-coated PIE OWG sensor is suitable for selective detection of the DCP vapor.

In this section, the response of the OWG sensor to strong acids was also investigated by injecting saturated steam of hydrochloric acid (HCl) into the sample chamber. It could be found that the response of the OWG sensor to saturated steam of HCl is similar to that of the DCP vapor, and it is difficult to differentiate the response to saturated steam of HCl and DCP vapor. However, compared with hydrochloric acid, there was a stronger response of the OWG sensor to DCP with the same concentration. Therefore, when the detection of DCP carried on, the interference of strong acid should be eliminated.

Regarding the OWG sensor, the sensing film’s optical properties (absorbance, scattering, refractive index, and fluorescence/luminescence) are the main factors that affect its sensitivity. When a thin film is coated on the surface of the waveguide layer, the evanescent wave produced by the 633 nm light wave traveling through the waveguide layer would come into the thin film. The change of OWG sensor’s output light intensity caused by the variation of optical properties is related to the analyte concentration, and this provides the foundation for the sensor response. In this paper, the variation in CV-SiO_2_ gel film’s absorbance/transmittance induced the changes in intensity of the output light when the sensing device was exposed to the DCP vapor.

## 4. Conclusions

In summary, an OWG sensor locally coated with CV-SiO_2_ gel film had been developed for DCP vapor detection. The _Δ_*I* of the OWG sensor significantly increased with the augment of DCP concentration, and it exhibited good linearity between the response intensity of the OWG sensor and the natural logarithm concentrations of the DCP vapor in a range from 1.68 × 10^−9^ to 26.32 × 10^−6^ (volume fraction). Clear interference with the DCP detection was observed in the presence of other common solvents, benzene series, phosphorus-containing reagents, acid, and basic gas. Meanwhile, in comparison with previous works [1,38] and our recent paper [39], this OWG sensor possesses a lower quantitative volume fraction of 1.68 × 10^−9^ for the DCP vapor. Compared with the traditional method such as chromatography, ion migration spectrometry, and Raman, the structure of the OWG sensor we proposed in this article is simple, low-cost, and reliable. Especially, the OWG sensor does not need carrier gas and an ion source. Its optical structure is simple and adjustable as well. Moreover, the OWG sensor possesses satisfactory selectivity and sensitivity for the detection of DCP gas. Its sensor chips are easy to prepare and the equipment is also easy to maintain. Overall, the OWG sensor proposed could provide quantitative, real-time, fast, and accurate measurement results for the detection of the DCP vapor, and it would be a probable and practical approach used for widespread applications in food safety, environmental monitoring, homeland security, and poisonous gas detection.
